# Isolation of *Pseudomonas aeruginosa* in Stable Chronic Obstructive Pulmonary Disease Patients—Should We Treat It?

**DOI:** 10.3390/jcm12155054

**Published:** 2023-08-01

**Authors:** Jose Daniel Gomez-Olivas, Grace Oscullo, Miguel Angel Martinez-Garcia

**Affiliations:** 1 Servicio de Neumología, Hospital Universitario y Politécnico La Fe, Avenida Fernando Abril Martorell 2026, 46026 Valencia, Spain; jdaniel365@hotmail.com (J.D.G.-O.); graceoscullo@gmail.com (G.O.); 2 CIBERES de Enfermedades Respiratorias, ISCIII, 28029 Madrid, Spain

Chronic obstructive pulmonary disease (COPD) is one of the most frequent inflammatory diseases of the airways [1,2,3]. It is characterized by chronic airway inflammation with a neutrophil predominance and a variable number of eosinophils and mononuclear cells, produced mainly by tobacco smoke and other environmental causes, in genetically predisposed individuals [4,5]. Even if it is treated or smoking is abandoned, this inflammation usually remains to a certain extent, and may increase as a consequence of the exacerbations that usually appear throughout the natural history of the disease, producing the progressive deterioration of the patient [6]. This chronic inflammation favours damage to the local defence mechanisms against infection, and therefore increases the probability of bronchial infection by potentially pathogenic microorganisms (PPM), particularly bacteria. In fact, some PPM have been isolated from 8–43% (depending on the study) of the respiratory samples (usually sputum) of patients with COPD, even in a clinically stable phase, the most frequent being *Haemophilus influenzae*, *Streptococcus pneumoniae*, *Moraxella Catarhhalis*, *Pseudomonas aeruginosa* and other Gram-negative rods [7,8,9,10].

Sometimes, these isolates tend to appear sporadically and even singly, without producing significant clinical changes in the patient. However, on other occasions, isolation of the same PPM is perpetuated over time—a circumstance that is called chronic bronchial infection (CBI) [11]. There is no established definition of CBI in patients with COPD, but it increases the possibility of a negative impact on the clinical severity or prognosis of the patient. In fact, several studies have related the presence of a CBI in COPD patients to greater local and systemic inflammation, greater clinical severity, poorer quality of life, an increase in the number of cardiovascular events, an accelerated loss of lung function, a greater number and severity of exacerbations and even higher mortality [7,8,9,10,11].

Among the PPM that can be isolated in the natural course of COPD, *Pseudomonas aeruginosa* (PA) has perhaps caused the most controversy in terms of its management. In other airway diseases such as bronchiectasis or cystic fibrosis, PA is one of the most frequently isolated PPM [12,13]. The isolation of PA (even for the first time) has been clearly related to a rapid deterioration and poorer vital prognosis of the disease [14]; accordingly, the main therapeutic guidelines recommend antibiotic and/or anti-inflammatory treatments after even the first isolation of PA, in an attempt to eradicate it or at least reduce its bacterial load in the airways for as long as possible [15,16,17]. In patients with COPD, however, the scientific evidence on the impact of PA isolation (single or repeated) is not as conclusive, so its therapeutic management remains controversial.

Cross-sectional studies have concluded that PA can be isolated in up to 3–15% of COPD patients, especially those with greater severity, those with an exacerbator phenotype, those with an active smoking habit, after admission to an Intensive Care Unit, after the administration of several courses of antibiotics or systemic corticosteroids, and those with the concomitant presence of bronchiectasis [18]. As regards the specific impact of PA on the prognosis of patients with COPD, one recent meta-analysis that included more than 23,000 patients (derived from cohort studies in the majority of cases) who were followed for 1 to 7 years concluded that the adjusted risk of death was almost double in those patients with COPD and isolation (single or repeated) of PA, compared to those in whom it had never been isolated (HR 1.95; 95% CI: 1.34 to 2.84) [18]. In this respect, another study further concluded that the risk of death was more than tripled (HR 3.06; 95%CI: 1.8 to 5.2) if PA could be isolated multiple times (CBI situation) [19]. In fact, PA is the only PPM that has been shown to be associated with an increase in mortality in COPD independently of other confounding variables [18]. As regards any increase in the number and severity of exacerbations, the results are more mixed, although most studies agree that PA is associated with such an increase [18]. Finally, there is hardly any literature available on PA’s effect on lung function or its evolution, or on patients’ quality of life [18]. Significantly, it is still not known whether PA is a marker of COPD severity (i.e., whether its isolation occurs as a consequence of the advanced stage of the disease) or whether PA itself causes the accelerated deterioration of COPD. It is very probable that both circumstances coexist, since PA is a pathogen that usually takes advantage of extensive disruption and damage to defence mechanisms in order to infect the airways, although more rapid deterioration of COPD has also been observed after a first isolation of PA (since this first isolation is usually performed with sputum samples; however, there is no guarantee that the PA has not already been infecting the airways of a COPD patient for some time) [20].

However, the main question that clinicians face is: what should I do when faced with PA isolation in a COPD patient? Although the current scientific evidence does not permit any definitive answer to this question, some steps do seem recommendable: first, the performance of high-resolution computed tomography (HRCT) to rule out the presence of bronchiectasis (which is considered a treatable trait in COPD); second, a clinical assessment of the patient (especially the characteristics of the sputum and the number and severity of exacerbations); and, finally, microbiological monitoring of the patient’s sputum (to assess the persistence of PA over time), as already recommended by some international COPD guidelines [5,21]. These steps open up various therapeutic scenarios, in the opinion of the authors of this editorial. 1. A patient with symptomatic COPD and bronchiectasis (BCOS). In this case, there is considerable unanimity that it is necessary to follow the recommendations of the bronchiectasis guidelines regarding the necessary antibiotic and/or anti-inflammatory treatment after the first PA isolation [15,16,17]. 2. A patient with COPD and isolated PA (single or multiple) but without bronchiectasis. In this case, the recommendation would be the treatment of PA infection in the case of patients with multiple exacerbations or clinical deterioration secondary to this infection, with earlier and more forceful treatment more recommendable in the case of CBI [7,8]. 3. A patient with asymptomatic COPD and single or multiple isolations of PA. In this case, it is possible that close clinical and microbiological monitoring of the patient is the most suitable approach, with treatment applied in the event of clinical deterioration or an increase in the number of exacerbations, especially in situations of CBI [7,8].

However, it is important to emphasize that the steps proposed above are no more than recommendations by some experts, with a low degree of scientific evidence (except when bronchiectasis coexists, where the amount of evidence is greater). Similarly, there is no evidence as regards the optimal treatment. There have been no specific studies in patients with COPD and bronchial PA infection on the effect of inhaled antibiotics or macrolides (as immunomodulators), although it is true that macrolides have been shown to significantly reduce the number of exacerbations in patients with both bronchiectasis [22] and COPD (without differentiating whether or not CBI infection existed) [23]. Treatment with inhaled antipseudomonic antibiotics would be another option [24], although this remains more controversial due to the lack of studies in COPD to date. Finally, special mention should be made of treatment with inhaled corticosteroids (ICS) in these patients; although their strong anti-inflammatory effect is known, as is their immunosuppressive effect, which could produce a deleterious impact on patients with PA infection. Once again, the scientific evidence is scarce, so some authors have recommended offering ICS to patients with COPD with multiple exacerbations and peripheral eosinophilia only at the lowest possible effective dose, precisely to avoid these potential deleterious effects [25].

In short, although the isolation of PA (and other PPM) is often found in patients with COPD and has given rise to controversy, the existing evidence in this regard is very scarce, and so this editorial only seeks to reflect the personal opinion of its authors (shared by other experts) on the management of this situation. Further studies are needed, however, especially on the technical development, analysis and clinical applicability of the changes in the lung microbiome profile (dysbiosis) instead of the classical microbiological techniques of pathogenic microorganisms isolations from respiratory samples [26,27], as well as clinical trials that would definitively demonstrate the effect of existing treatments and other promising ones, such as neutrophil elastase inhibitors [28,29], or the use of big data analysis [30,31] to better assess the impact of single or repeated isolations of PA in COPD patients.
